# Can Ultrasound‐Guided in‐Plane Puncture Technique Enhance the Precision of Femoral Artery Access? The Randomized PARFEM Trial

**DOI:** 10.1002/ccd.31733

**Published:** 2025-07-30

**Authors:** Wolfram Voelker, Maren Lachmann, Guelmisal Gueder, Ahmed Bayik, Margarete Heinrichs, Ralph Kickuth, Wolfgang R. Bauer, Bjoern Lengenfelder, Peter Nordbeck, Stefan Frantz, Stefan Störk

**Affiliations:** ^1^ Department of Internal Medicine I—Cardiology University Hospital Würzburg Würzburg Germany; ^2^ Caritas‐Krankenhaus Bad Mergentheim Bad Mergentheim Germany; ^3^ Department of Diagnostic and Interventional Radiology University Hospital Würzburg Würzburg Germany; ^4^ Department Clinical Research & Epidemiology, Comprehensive Heart Failure Center University Hospital Würzburg Würzburg Germany

**Keywords:** femoral access, high stick, in‐plane, retroperitoneal hemorrhage, ultrasound‐guidance

## Abstract

**Background:**

Ultrasound‐guided puncture of the common femoral artery (CFA) is assumed to be more precise than conventional techniques without ultrasound. However, previous comparative studies have shown partly contradictory results with regard to success of ultrasound‐guided puncture so that this method is not yet standard in transfemoral cardiac catheterization.

**Aims:**

The PARFEM trial was performed to investigate whether an ultrasound‐assisted in‐plane technique using a needle guide could improve the precision of CFA puncture.

**Methods:**

The study was conducted in 286 patients undergoing transfemoral cardiac catheterization. Patients were randomized 1:1, and femoral artery puncture was performed either using an ultrasound‐guided in‐plane technique with fluoroscopic marking of the center of the femoral head or a conventional approach. The primary endpoint was successful puncture of the CFA with the first attempt. In addition, the influence of the examiners' level of experience on puncture results was investigated.

**Results:**

Primary successful puncture of the CFA was achieved more frequently in the ultrasound group than in the control group: 79.6% versus 55.6%, odds ratio 3.25 [95% CI: 1.92−5.23], *p* < 0.001. This favorable effect of ultrasound guidance was independent of the examiners' level of experience. The success rate of ultrasound‐guided sheath placement within the CFA was 97.2%, compared to 77.8% in the control group (*p* < 0.01).

**Conclusions:**

The PARFEM trial demonstrated that in‐plane ultrasound guidance can improve the precision of femoral puncture and reduce the occurrence of inadequate sheath placements. The results support the incorporation of this technique into standard practice for guiding transfemoral puncture.

**Trial Registration:**

ClinicalTrials.gov Identifier: NCT06065943.

**Abbreviations**
CFA: common femoral artery
FH: femoral head

## 1 Introduction

Several studies have demonstrated that cardiac catheterization via the radial artery significantly reduces vascular complications compared to the femoral approach [1, 2]. Therefore, transfemoral catheterization is now employed only in select groups of patients such as those with severe radial spasm, pronounced vascular tortuosities, or those requiring large‐lumen sheaths.

When a transfemoral access has to be performed, a correct puncture technique is mandatory. Selecting the correct puncture height is crucial to minimize the risk of complications. While a puncture above the inguinal ligament significantly increases the risk of retroperitoneal bleeding [3, 4, 5], a puncture below the femoral bifurcation elevates the likelihood of post‐interventional bleedings or pseudoaneurysms [6, 7, 8]. An ultrasound‐guided puncture technique enables a targeted puncture of the femoral vessel, that is, a puncture within a suitable, calcification‐free vessel segment. In contrast to the classical technique guided by palpation and fluoroscopy, where the puncture is performed virtually “blind,” the ultrasound‐supported procedure allows the vessel to be punctured “under vision.” Especially for less experienced operators, ultrasound is aimed at minimizing complications and ensuring safer procedures. In the meantime, numerous studies [9, 10, 11, 12, 13, 14, 15] have assessed whether ultrasound‐guided puncture is more accurate and causes fewer complications than the conventional method. To date, no randomized study has demonstrated the superiority of ultrasound‐guided femoral puncture. The three major multicenter trials—FAUST [9], SURF [13], and UNIVERSAL [15] adopted distinct primary objectives, choosing either a procedural (“successful common femoral artery cannulation”) or a combined clinical endpoint. Notably, the primary endpoint analyses in all three trials were unequivocally negative. Only some meta‐analyses have indicated potential advantages of the ultrasound‐guided puncture technique [16, 17]. Due to these mixed results, ultrasound‐guided puncture has not yet become the standard practice in transfemoral cardiac catheterization [18].

One reason for the partly negative results could be the “out‐of‐plane” technique of ultrasound guidance, a widely used technique that was also applied in the cited studies, see the schematic representation of the method in Figure S1. In contrast, the PARFEM trial (ULTRASOUND‐GUIDED IN‐**P**L**A**NE PUNCTU**R**E OF THE **FEM**ORAL ARTERY) used an “in‐plane” ultrasound guidance technique (Figure S1).

The objective of the PARFEM trial was to assess whether this technique can increase the precision of femoral artery puncture as a prerequisite for reducing vascular complications.

## 2 Methods

### 2.1 Study Design

The PARFEM trial was a randomized controlled trial using an ultrasound‐assisted in‐plane puncture technique combined with fluoroscopic marking of the center of the femoral head. The trial was approved by the Ethics Committee of the University Hospital Würzburg, and all patients provided written informed consent before any study procedure.

The procedures were performed by 23 medical staff members of the Department Internal Medicine I of the University Hospital Wuerzburg, including 9 consultant cardiologists and 14 cardiology fellows. Based on their level of experience, they were divided into two groups: Group 1 consisted of 13 examiners with moderate experience (50−500 transfemoral examinations/interventions performed), while Group 2 included 10 examiners with extensive experience (> 500 transfemoral catheter examinations/interventions performed). The first group was termed “non‐experts,” whereas the second was labeled “experts.” Each of the examiners performed an average of 12.4 (range: 1–49) examinations within the PARFEM trial.

### 2.2 Eligibility Criteria and Randomization

Patients who were scheduled to undergo an elective transfemoral procedure were eligible for inclusion into the study. Patients presenting with acute coronary syndrome were excluded as were patients with peripheral artery disease with no palpable pulse.

To investigate the impact of the individual catheter expertise on the primary endpoint, patients were stratified based on the experience level of the examiners. For each patient included in the study, the responsible examiner—assigned to either Group A or Group B—was chosen based on the anticipated complexity of the planned procedure and in accordance with the clinic's standard protocols. Upon determining the examiner's group affiliation, a stratified randomization was carried out 1:1 using a computer algorithm. Each patient was assigned to either “ultrasound‐guided puncture” or “conventional palpation and fluoroscopy‐assisted puncture.”

Before the examination of their first study patient, the participating physicians were familiarized with each step of the study protocol by the principal investigator.

### 2.3 Ultrasound‐Guided Puncture

**Figure 1:**
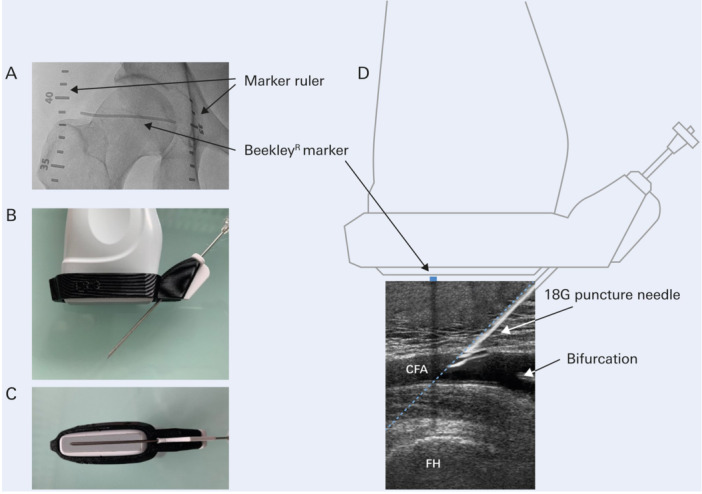
Ultrasound‐guided in‐plane puncture as used in the PARFEM trial. An ultrasound guided in‐plane puncture technique was used in which the CFA and the femoral head (FH) were visualized in the longitudinal plane (D). The center of the FH served as the upper border, and the bifurcation of the CFA as the lower border of the targeted puncture height. Within this zone, a suitable site for vessel puncture was identified. To ensure that the needle was continuously visualized in the displayed ultrasonic plane, a needle guide was used (B, C). Utilizing a displayed 45‐° reference line (D), the needle was advanced under ultrasound guidance. As an additional orientation landmark, a Beekley marker was placed horizontally at the level of the center of the FH with the aid of two rulers (A). The marker created a characteristic linear acoustic shadow (D), which helped to select the correct puncture height and to avoid “high sticks.” CFA = common femoral artery; FH = femoral head. [Color figure can be viewed at wileyonlinelibrary.com]

In the patients, who were assigned for ultrasound guided puncture, two sterile marker rulers (Glow'N Tell Tape, LeMaitre Vascular, Burlington, MA, USA) were taped in the longitudinal direction lateral and medial of the femoral head. Then, an X‐ray image capturing both the femoral head and the marker rulers was performed. Using this image, an echo‐tight tape (Beekley, CT‐SPOT 118, Bristol, CT, USA) was fixed between the two rulers in such a way that it traversed horizontally through the center of the femoral head (Figure 1A and Video S9).

An ACUSON‐Freestyle ultrasound device (Siemens, Forchheim, Germany), embedded into the X‐ray‐system (Azurion, Philips, Best, The Netherlands), was used. The corresponding L 8‐3 vascular transducer was first packaged sterile (Snap‐fit, Civco, Coralville, Iowa, USA) and then fitted with a sterile needle guide (GalloPrint, Hettstadt, Germany) (Figure S2). This needle guide is a 3D‐printed disposable product through which an 18 G puncture needle (VascularSono, 10 cm, Pajunk, Geisingen, Germany) can be advanced in‐plane at an angle of 45° (Figure 1B,C). For very slender patients, a 30‐° needle guide was available.

The transducer was positioned to obtain a longitudinal view of the femoral artery. First, the femoral bifurcation was identified as the imaginary lower limit of the puncture site. If the bifurcation could not be clearly identified, the vessel was additionally visualized in an oblique and short axis view. Then, the center of the femoral head was identified by ultrasound, serving as the upper limit of the targeted puncture segment. The reliability of this visualization depended on the quality of the femoral head ultrasound image, which was assessed and documented by the examiner.

The following definitions were applied:

1. Good scan quality: The femoral head could be fully visualized.
2. Moderate scan quality: The femoral head could be partially visualized.
3. Poor scan quality: The femoral head could not be visualized.

Based on the initial ultrasound scan‐in case of uncertainties, supported by the Beekley marker‐ a calcification‐free site was identified within the target puncture segment, preferably at the level of the lower half of the femoral head. Then, the vessel was adjusted longitudinally so that the 45‐° needle guidance line aligned with the selected target site of the vessel (Figure 1D). Vertical placement of the ultrasound probe ensured a clear and sharp depiction of the endothelium, indicating central puncture positioning. The needle was advanced under continuous ultrasound imaging. Penetration of the anterior vessel wall was performed slowly to avoid perforation of the posterior vessel wall; the intravascular position of the needle tip was usually clearly recognized. Vessel entrance was typically accompanied by pulsatile blood backflow through the needle. A 0.035‐in. guidewire was inserted through the needle under ultrasound guidance. If resistance was encountered, the needle tip was slightly repositioned to allow unimpeded insertion of the wire. A femoral sheath (Radifocus, Terumo, Tokyo, JP) was finally placed.

### 2.4 Control Group

To select the appropriate femoral puncture site in the control group, the common anatomical landmarks were identified. Considering these landmarks, the puncture was performed using an 18 G needle at the site of the strongest palpable pulse. The examiner decided whether to use fluoroscopy to facilitate the process of determining the puncture height. After insertion of the 0.035” wire, a femoral sheath was advanced.

### 2.5 Rotational Angiography

The position of the sheath was finally documented by angiography. A “single axis” rotational angiography of the femoral vessel was performed. For this purpose, the C‐arm was rotated at a speed of 50°/s from RAO 40 to LAO 40 (Figure S3) without cranial or caudal angulation. Automatic (ACIST CVi) or manual contrast injection was performed through the side port of the sheath and was started immediately before C‐arm rotation.

### 2.6 Removal of the Sheath

In all four groups, the sheath was removed at the end of the procedure. It was left at the discretion of the examiner to use a vascular closure system or manual compression only. If rotational angiography showed the sheath distal to the bifurcation, the use of Angioseal (Terumo, Tokyo, JP) or Proglide (Abbott Vascular, Redwood City, Ca) was avoided.

### 2.7 Assessment of the Rotational Angiograms

The rotational angiograms were evaluated by two experienced team members. To view the groin vessels from different projections, the angiograms were replayed multiple times. Using a projection in which the inferior epigastric artery was shown, the lowest point of this vessel was identified, which marks the upper boundary of the CFA [4]. In a further projection, the bifurcation of the CFA was identified. Finally, a projection was selected in which the entry of the sheath into the vessel was most clearly visible (Figure S3). Cannulation of the CFA was considered successful if the puncture site was located distal to the inferior loop of the inferior epigastric artery and proximal to the femoral bifurcation. If the vascular entry of the sheath was at or above the inferior epigastric artery or distal to the bifurcation, the cannulation was considered “too high” (located in the external iliac artery) or “too low” (located in the superficial or deep femoral artery) (Figure S4).

### 2.8 Endpoints

The successful puncture of the CFA at the first attempt was defined as the primary endpoint. The basis for this approach was that achieving an initial successful puncture almost entirely prevents vascular complications related to injuries of adjacent structures. Secondary endpoints included “first‐pass success,” defined as the percentage of patients with a primary successful arterial puncture, regardless of the puncture site. Furthermore, the number of attempts, the percentage of patients in whom the sheath was adequately placed in the CFA, the time to achieve femoral access, the rate of inadvertent venipunctures, and the occurrence of bleeding complications requiring vascular intervention or surgery were documented. Subgroup analysis was conducted based on the following prespecified parameters: age (≥ 75 years vs. < 75 years), sex (male vs. female), body mass index (BMI; weight in kg divided by height in meters squared; ≥ 30 vs. < 30), type of coronary catheterization (PCI vs. angiography alone), sheath size (≥ 6 F vs. < 6 F), right heart catheterization (performed vs not performed), and closure device (used vs. not used). Additionally, two post hoc‐defined subgroups were examined: femoral head image quality (Grade 1 vs. Grades 2 or 3) and ultrasound experience of the examiner (Level 1 vs. Level 2, Appendix S7).

### 2.9 Sample Size Calculation and Data Analysis

The sample size calculation for PARFEM was based on the first pass success rates reported in the Faust trial [9], where these rates were 82.7% in the ultrasound group and 46.4% in the control group. Our hypothesis was that in PARFEM at least the same first pass success rates could be achieved as reported in [9]. With improved technology (in‐plane, needle guidance, and fluoroscopic marking), we aimed to avoid punctures outside the CFA and selected “successful CFA puncture at the first attempt” as the primary endpoint. Assuming *α* = 0.05 and a power (1‐*β*) = 0.90, 105 patients per study arm were required to detect a difference in the primary endpoint if the success rate was 80% in the ultrasound arm and 60% in the control arm.

Data are described using count (percent), mean (SD), median (quartiles and range), as appropriate. Differences between groups were tested using the Mann−Whitney *U*‐test and Kruskal−Wallis test, as appropriate. *p* < 0.05 were considered statistically significant. No correction was made for multiple testing. Statistical analyses were performed using SPSS version 27. Study data were collected and managed using REDCap electronic data capture tools hosted by the University of Würzburg.

## 3 Results

### 3.1 Patient Population

The PARFEM trial enrolled and randomized 300 patients who underwent transfemoral cardiac catheterization at the University Hospital Wuerzburg between July 2022 and March 2024. During this period, a total of 4196 cardiac catheterizations/interventions were performed at the study site.

**Figure 2:**
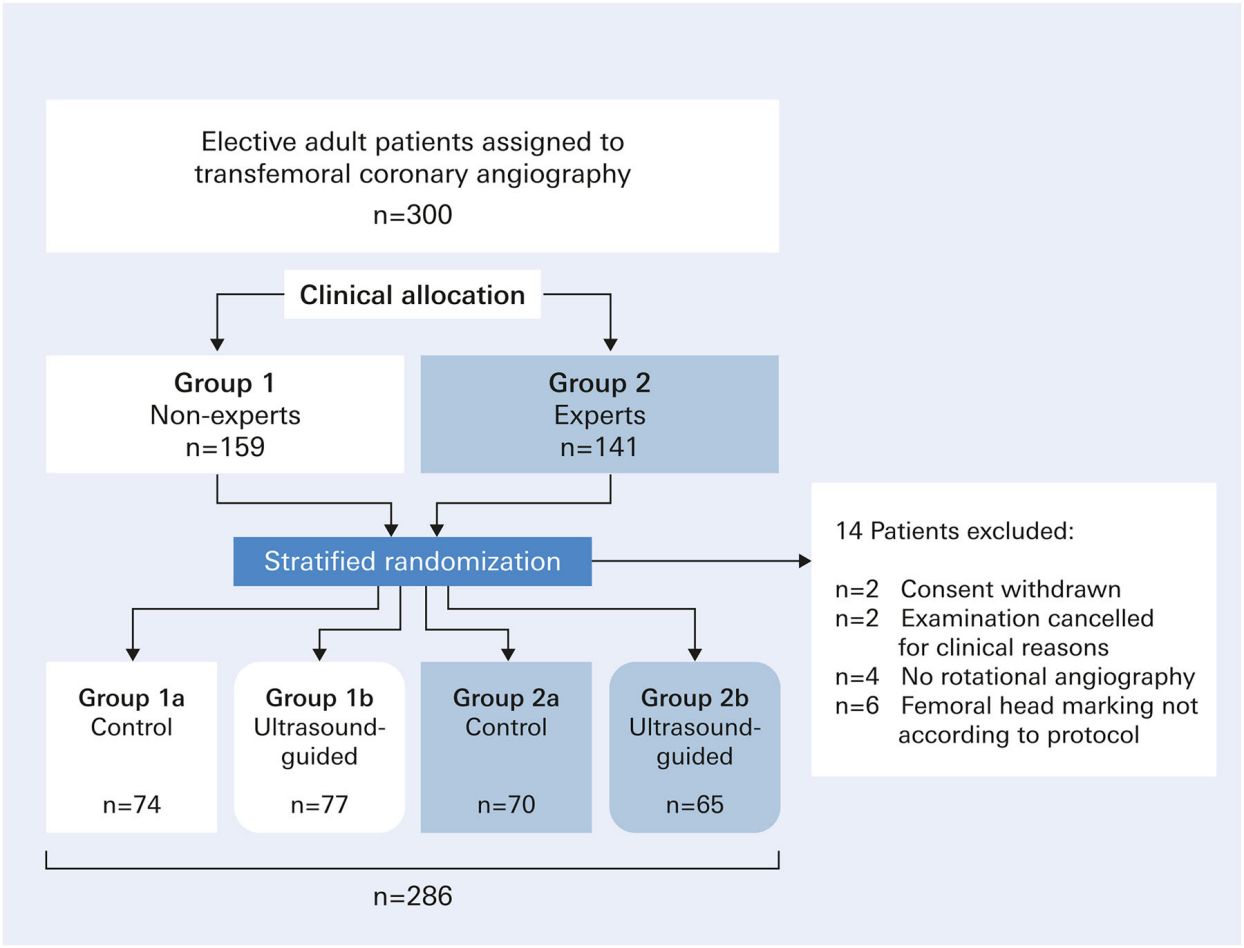
CONSORT diagram of the diagnostic PARFEM trial. [Color figure can be viewed at wileyonlinelibrary.com]

Fourteen patients were excluded from the study for various reasons (Figure 2). The remaining 286 patients (66% men, 34% women) had a mean age of 71.5 ± 10.5 years. Diagnostic coronary angiography was performed in 220 patients (77%), while 66 patients (23%) underwent PCI. Closure devices were placed in 211 patients (73.8%). The demographic and procedural parameters were similar between all four study groups (Table 1).

**Table 1:** Demographics and procedural characteristics of study participants (n = 286).

	Non‐Experts *N* = 151			Experts *N* = 135		
	Control *N* = 74	Ultrasound‐guided *N* = 77	*p* value	Control *N* = 70	Ultrasound‐guided *N* = 65	*p* value
**Demographics**						
Age (years)	72.0 (10.2)	70.5 (10.5)	0.447	71.8 (10.9)	71.9 (10.5)	0.946
Male sex	47 (63)	51 (66)	0.726	44 (63)	46 (71)	0.330
**Body mass index (kg/m²)**						
Mean	28.0 (4.4)	27.1 (4.5)	0.260	28.6 (6.4)	27.0 (4.2)	0.177
≥ 30 kg/m²	26 (35)	20 (26)	0.242	27 (39)	18 (28)	0.201
**PAD (Fontaine)**	2 (3)	6 (8)	0.163	5 (7)	4 (6)	0.330
**Chronic kidney dysfunction** a	15 (20.3)	17 (22.1)	0.786	18 (25.7)	18 (29.5)	0.628
**Procedure**						
Diagnostic	58 (78)	62 (80)	0.745	53 (76)	47 (72)	0.652
Intervention	16 (22)	15 (20)	0.745	17 (24)	18 (28)	0.652
Additional right heart catheter	20 (27)	24 (31)	0.576	19 (27)	25 (39)	0.161
**Anticoagulation**						
Heparin	74 (100)	77 (100)	—	70 (100)	65 (100)	—
P2Y12 inhibitor	11 (15)	10 (13)	0.712	8 (12)	7 (11)	0.880
GP IIbIIIa antagonist	0 (0)	0 (0)	—	1 (1)	0 (0)	0.333
**Sheath size**						
French 5	57 (77)	61 (79)	0.744	48 (69)	49 (76)	0.379
≥ French 6	17 (23)	16 (21)	0.744	22 (31)	16 (24)	0.379
**Closure device**						
Angioseal	8 (11)	5 (7)	0.344	16 (23)	9 (14)	0.178
Exoseal	51 (69)	47 (61)	0.310	39 (56)	33 (51)	0.565
Mynx	0 (0)	0 (0)	N/A	1 (1)	1 (2)	0.958
Prostyle	0 (0)	0 (0)	N/A	0 (0)	1 (2)	0.298

*Note:* Data are count (percent) or mean (SD), as appropriate.

Abbreviations: PAD = peripheral artery disease.

^a^ Creatinine > 1.3 mg/dL.

### 3.2 Primary Endpoint

**Figure 3:**
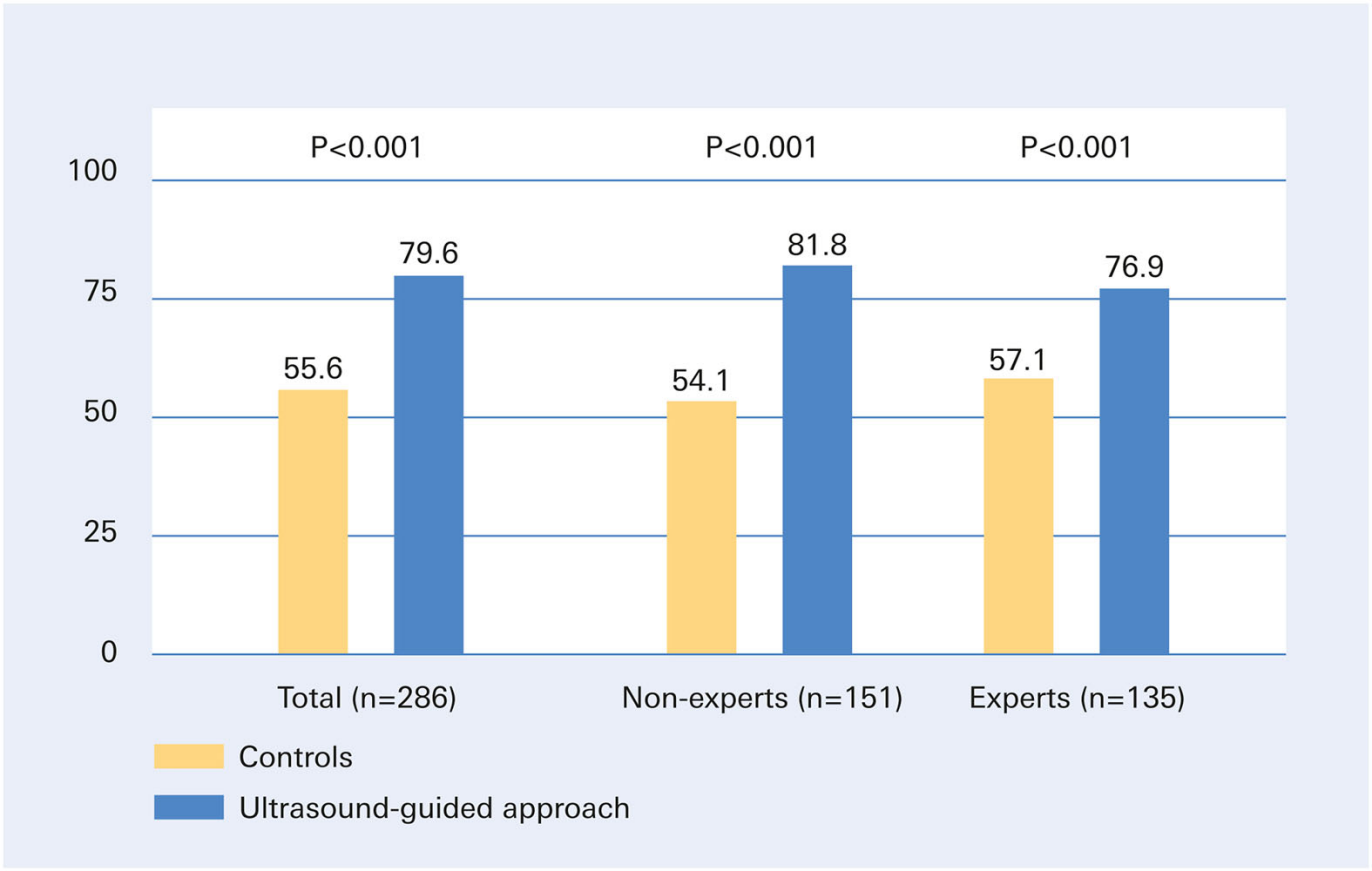
Successful puncture of the common femoral artery on the first attempt. Primary successful puncture of the common femoral artery (primary endpoint of the PARFEM trial) was achieved significantly more often in the 142 patients punctured with ultrasound guidance than in the 144 controls (left columns). The benefit of ultrasound‐guided puncture was independent of the examiners’ level of femoral puncture experience and was evident in both “non‐experts” (middle columns) and “experts” (right columns). Values expressed as percentages. [Color figure can be viewed at wileyonlinelibrary.com]

In the overall cohort, patients who underwent ultrasound‐guided puncture were significantly more likely to have a successful CFA puncture on the first attempt compared to those who did not (79.6% vs. 55.6%, odds ratio 3.25 [95% CI, 1.92−5.23], *p* < 0.001). This favorable effect of ultrasound guidance was achieved regardless of the experience level of the examiner and was observed in both “non‐experts” and “experts” (Figure 3 and Table 2).

**Table 2:** Intraprocedural and clinical outcomes.

Endpoint	Total group			Non‐experts			Experts		
	Control *N* = 144	Ultrasound‐guided *N* = 142	*p* value	Control *N* = 74	Ultrasound‐guided *N* = 77	*p* value	Control *N* = 70	Ultrasound‐guided *N* = 65	*p* value
**Primary endpoint**									
First‐pass CFA access	80 (55.6)	113 (79.6)	< 0.001	40 (54.1)	63 (81.8)	< 0.001	40 (57.1)	50 (76.9)	< 0.001
**Secondary endpoints**									
First pass arterial access	105 (73.4)	116 (84.1)	0.030	55 (75.3)	64 (84.2)	0.177	50 (71.4)	52 (83.9)	0.089
Successful CFA placement	112 (77.8)	136 (97.2)	< 0.001	57 (77.0)	75 (97.4)	< 0.001	55 (78.6)	61 (96.8)	0.002
High stick^a^	16 (11.1)	3 (2.1)	0.002	10 (13.5)	2 (2.6)	0.013	6 (8.6)	1 (1.6)	0.072
Low stick^b^	16 (11.1)	1 (0.7)	< 0.001	7 (9.5)	0	0.006	9 (12.9)	1 (1.6)	0.014
Unintended venipuncture	8 (5.6)	5 (3.5)	0.158	3 (4.1)	2 (2.6)	0.628	5 (7.1)	3 (4.6)	0.564
Attempts for arterial puncture	1.5 (1.1)	1.2 (0.7)	0.028	1.6 (1.4)	1.2 (0.5)	0.13	1.4 (0.7)	1.3 (0.8)	0.177
Time to access (s)	73 (109)	90 (108)	< 0.001	83 (129)	107 (122)	< 0.001	63 (83)	69 (84)	0.261
Patients requiring surgical or interventional treatment	3 (2.1)	1 (0.7)	0.321	1 (1.4)	1 (1.3)	0.977	2 (2.9)	0	0.170

*Note:* Data are count (percent), mean (SD), or median (quartiles), as appropriate.

Abbreviations: CFA = common femoral artery.

^a^ Puncture of external iliac artery.

^b^ Puncture of the superficial/deep femoral artery.

### 3.3 Secondary Endpoints

The first‐pass success rate was markedly higher with ultrasound guidance compared to conventional puncture (84.1% vs. 73.4%, *p* = 0.03), and was also evident in both “non‐expert” and “expert” examiners. Femoral angiography revealed that the sheath was located in the CFA in a total of 97.2% of the patients who underwent ultrasound‐guided puncture, compared to only 77.8% of patients in the control group (*p* < 0.01). Again, the beneficial effect of ultrasound guidance was not dependent on the level of experience. Concurrently, incorrect cannulations at or above the inferior epigastric artery occurred less often in the ultrasound‐guided group (2.1%) compared to controls (11.1%, *p* = 0.002). Similarly, too low cannulations (distal to the femoral bifurcation) also occurred less often with ultrasound‐guidance (0.7% vs. 11.1%, *p* < 0.001) (Table 2 and Central Illustration 1).

**Central Illustration 1:**
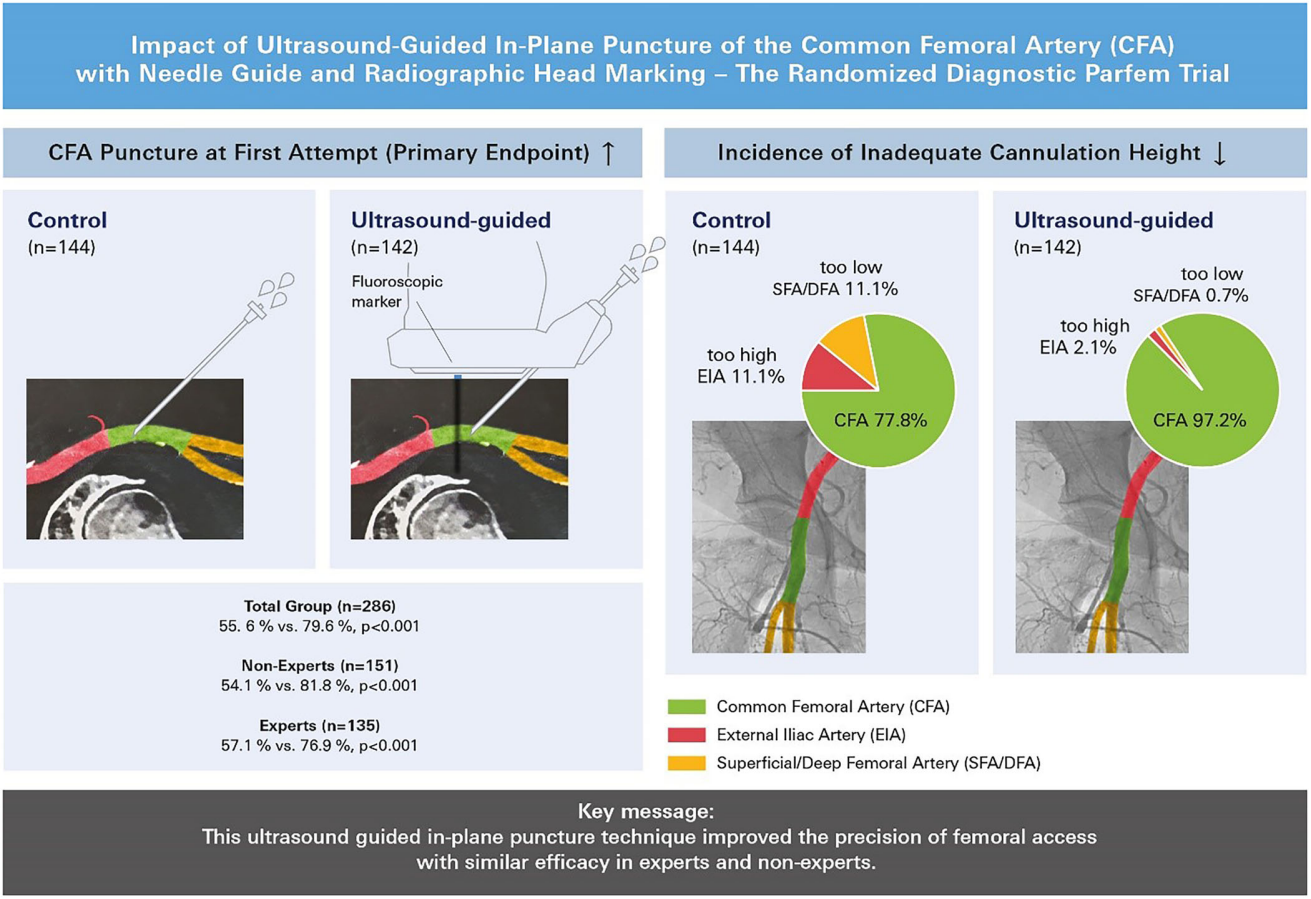
Ultrasound guided in‐plane puncture of femoral artery (PARFEM‐Trial). [Color figure can be viewed at wileyonlinelibrary.com]

The number of inadvertent venipunctures was not statistically different between the control group and the ultrasound‐guided group. However, there was a trend toward benefit of reduced inadvertent venipunctures with ultrasound in both the expert and non‐expert groups. (Table 2).

### 3.4 Major Complications

In the entire cohort, four patients experienced bleeding complications requiring vascular intervention or surgery. In the control group, three major vascular complications occurred, which required catheter intervention. Meanwhile, in the ultrasound‐guided group, only one patient suffered a major complication, a secondary hemorrhage from the CFA, necessitating surgical revision For details, Table S5.

### 3.5 Subgroup Analyses for the Primary Endpoint

**Figure 4:**
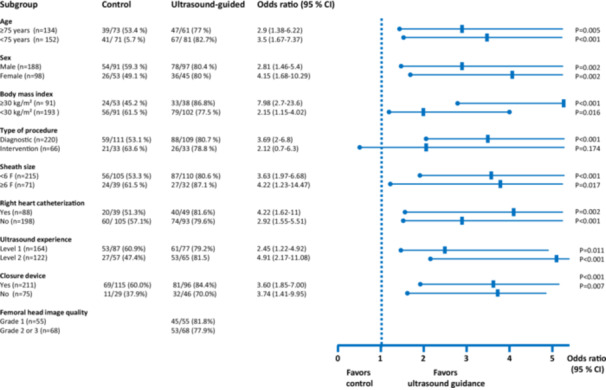
Subgroup analyses for the primary outcome. For definitions of femoral head image quality and ultrasound experience, refer to Methods. [Color figure can be viewed at wileyonlinelibrary.com]

Subgroup analysis revealed that primary successful puncture of the CFA was significantly more frequent with ultrasound guidance than in the control group, regardless of age, sex, weight, additional right heart catheterization, use of a closure system, or French size. Ultrasound guidance proved beneficial for patients with different BMI categories—both those with a BMI < 30 and those ≥ 30. However, the positive effect was more pronounced in patients with a BMI above 30. Furthermore, subgroup analysis showed that while ultrasound guidance had a significant benefit in diagnostic procedures, it was also present in PCI but did not reach statistical significance. Based on a questionnaire of the investigators (Appendix S7), two ultrasound competency level groups were formed. The subgroup analysis showed that investigators without prior experience in the in‐plane femoral puncture technique (Level 1) benefited in a similar way to those who already had experience with the method (Level 2) (Appendix S8). Femoral head scan quality did not affect the favorable impact of ultrasound guidance on the primary endpoint: Sheath placement in the CFA was successful in 53 of 68 patients with moderate or poor femoral head visualization (77.9%) versus in 45 of 55 patients with good image quality (81.8%) (Figure 4).

## 4 Discussion

The PARFEM trial demonstrated that ultrasound guidance can improve the rate of successful primary CFA punctures. The study also clearly demonstrated that the beneficial effect of ultrasound guidance was independent of the examiners' catheter experience and could therefore be achieved by both physicians with extensive expertise (“experts”) and those without this competence (“non‐experts”). Most randomized trials of ultrasound guidance for femoral puncture have focused on “first‐pass success” [9, 10, 11, 13, 14, 15], which is easy to measure because sheath angiography is not required. In four studies [9, 13, 14, 15], this parameter improved with ultrasound, similar to the findings in PARFEM. However, this parameter offers limited clinical insight, as a “successful puncture” does not necessarily indicate that this puncture has been perfomed at the correct level namely in the target vessel (CFA).

The PARFEM trial demonstrated a significantly higher success rate of CFA cannulation with ultrasound guidance compared to control (97.2% vs. 77.8%). In contrast, three of the four studies mentioned above [9, 11, 15] did not show such an increase in this parameter with ultrasound guidance. It is important to mention that in the PARFEM trial ultrasound guidance almost completely prevented sheath placement above the CFA: whereas the frequency of so‐called “high‐sticks” was 11.1% in controls, it was only 2.1% with ultrasound guidance (Central illustration 1). This beneficial effect, which was not observed in Faust (9) or Universal (15) (Table S6), may be attributed to the specific puncture technique in PARFEM.

### 4.1 Needle Guidance Technique

In contrast to Faust [9] and Universal [15], both of which used an out‐of‐plane puncture technique, the PARFEM trial used an ultrasound‐guided in‐plane puncture of the CFA (Figure S1). This technique offers several advantages: The needle tip can be continuously visualized as it is advanced. This allows the vessel to be punctured under direct real‐time visualization, avoiding the risk of puncturing the posterior wall. Additionally, the longitudinal orientation of the ultrasound probe visualizes the femoral head, making it easier to determine the correct puncture height. For puncture planning, a guiding line can be displayed on the screen, illustrating the intended needle's trajectory to the appropriate vessel segment (Figure 1). Finally, in‐plane imaging, can be used to monitor unobstructed wire advancement.

A significant drawback of the in‐plane puncture technique is the difficulty in aligning the needle with the ultrasound image. Less experienced examiners find it difficult to maintain visual control of the needle throughout the procedure. Maximum efficacy and minimal risk of failed puncture can only be achieved when the needle tip is continuously visible. In PARFEM, a needle guide was used for in‐plane ultrasound guidance, eliminating the need for manual alignment. This made the procedure intuitive and easy to learn. The positive impact of a needle guide on puncture outcomes has been demonstrated in other studies [19, 20].

A limitation of the in‐plane technique is the challenge of aligning with the bifurcation of the CFA when the superficial femoral artery and the deep femoral artery run side by side rather than one behind the other. In these cases, an additional oblique or short axis view is necessary to identify the bifurcation.

The ultrasound‐guided puncture technique has often been criticized for requiring more time than the conventional procedure. However, this assumption is contradicted by the results of Sorrentino et al. [16] who showed in a meta‐analysis of six randomized trials that the average “time to access” for ultrasound‐guided transfemoral puncture was lower than in the control group (172 ± 125 s vs. 203 ± 180 s, *p* < 0.01). In the PARFEM trial, the duration for both the control and ultrasound groups was shorter than the respective times reported in the meta‐analysis. The time for X‐ray imaging and applying the Beekley marker was excluded. The PARFEM trial used an ultrasound device that was integrated into the cathlab imaging system, eliminating the need for time‐consuming transport and start‐up of the ultrasound machine.

### 4.2 Fluoroscopic Marking of the Center of the Femoral Head

In PARFEM, the visualization quality of the femoral head was classified as moderate or poor in 68 out of 123 patients (55%). Subgroup analysis showed that ultrasound guidance still improved the primary successful CFA cannulation rate, even in patients with impaired femoral head visualization. It is likely that in these cases the examiners were guided by the ultrasound shadow of the Beekley‐line, marking the center of the femoral head.

### 4.3 Rotational Angiography

Unlike previous studies [9, 11, 14, 15], which assessed the sheath position using conventional femoral angiography, the PARFEM trial used rotational angiography for this purpose. This multiplanar imaging allows better delineation of individual anatomical landmarks and the height of sheath entry. It is superior to conventional sheath angiography with its intrinsic limitations [4, 21].

### 4.4 Limitations

Several clinical studies have demonstrated that the risk of retroperitoneal bleeding increases with a puncture at or above the level of the inferior epigastric artery [4, 22, 23, 24]. Consequently, the PARFEM trial used the inferior loop of the inferior epigastric artery as the upper limit of the target vessel (CFA). However, CT angiographic studies indicate that this loop does not always align precisely with the level of the inguinal ligament [25, 26]. In principle, the in‐plane puncture method can also be used without X‐ray‐labeling of the femoral head center. However, it is not possible to reliably determine what the results of this study would have been without the use of the Beekley marker. A randomized comparison between ultrasound‐guided puncture with and without femoral head marking would have been the best way to address this question. Group 1 (“Non‐Experts”) consisted of individuals with moderate experience. Absolute beginners who had performed fewer than 50 transfemoral catheterizations were not included. It is quite conceivable that they might have particularly benefited from ultrasound guidance. The study was conducted as a single‐center study. Due to organizational reasons, a multicenter trial was not feasible. The reviewers who assessed the post‐puncture angiograms could not be blinded, as the Beekley marker was visible in every rotational angiogram of patients who had undergone ultrasound‐guided puncture. The complication rates were low in both groups, but there was a trend toward benefit of ultrasound guidance. However, due to its relatively small number of patients, the PARFEM trial cannot claim to make reliable statements about the clinical outcome of ultrasound‐guided femoral puncture. Finally, in PARFEM, no large bore interventions were included, where a precise technique is most likely of particular clinical importance [27, 28].

### 4.5 Conclusions

In the randomized PARFEM trial, an ultrasound‐guided in‐plane puncture technique using a needle guide, combined with fluoroscopic marking of the center of the femoral head improved the precision of femoral puncture and reduced the rate of inadequate cannulation. The beneficial effect was achieved regardless of the examiners' prior experience. The findings strongly suggest that in‐plane ultrasound guidance is a valuable approach for femoral artery puncture.

## Conflicts of Interest

The authors declare no conflicts of interest.

## Supporting information


**Supporting Information Figure 1**. Out‐of plane vs. in‐plane puncture technique (as used in PARFEM).

**Supporting Information Figure 2**. Image of the transducer with the mounted needle guide.

**Supporting Information Figure 3**. Schematic illustration of rotational angiography.

**Supporting Information Figure 4**. Examples of proper (left) and inadequate cannulation heights.

**Supporting Information Table 5**. Clinical course of patients with bleeding complications.

**Supporting Information Table 6**. Angiographic results: Faust vs. Parfem resp. Universal.

**Supporting Information Appendix 7**. Questionnaire on prior ultrasound experience.

**Supporting Information Appendix 8.** Influence of ultrasound experience on primary endpoint.

**Supporting Information Video 9**. Step‐by‐step approach of ultrasound‐guided in‐plane puncture.
