# Supplementary figures and images for: Can Ultrasound‐Guided in‐Plane Puncture Technique Enhance the Precision of Femoral Artery Access? The Randomized PARFEM Trial
Source: Catheter Cardiovasc Interv. 2025 Jul 30;106(4):2252–62. doi: 10.1002/ccd.31733 (PMC12509261; doi:10.1002/ccd.31733)

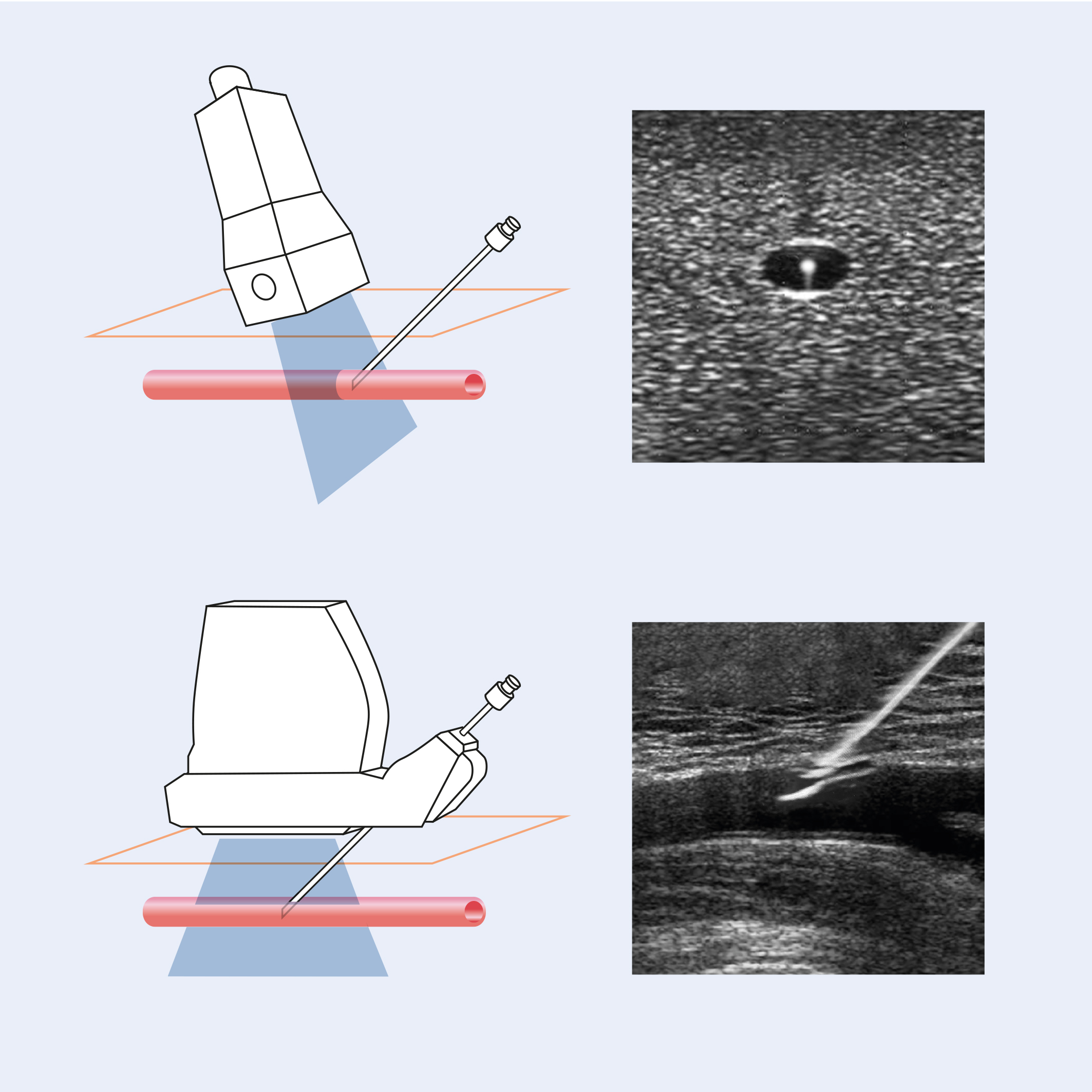

Supplement: Supplementary file 1 — Supporting Information Figure 1. Out‐of plane vs. in‐plane puncture technique (as used in PARFEM). [file CCD-106-2252-s003.jpg]

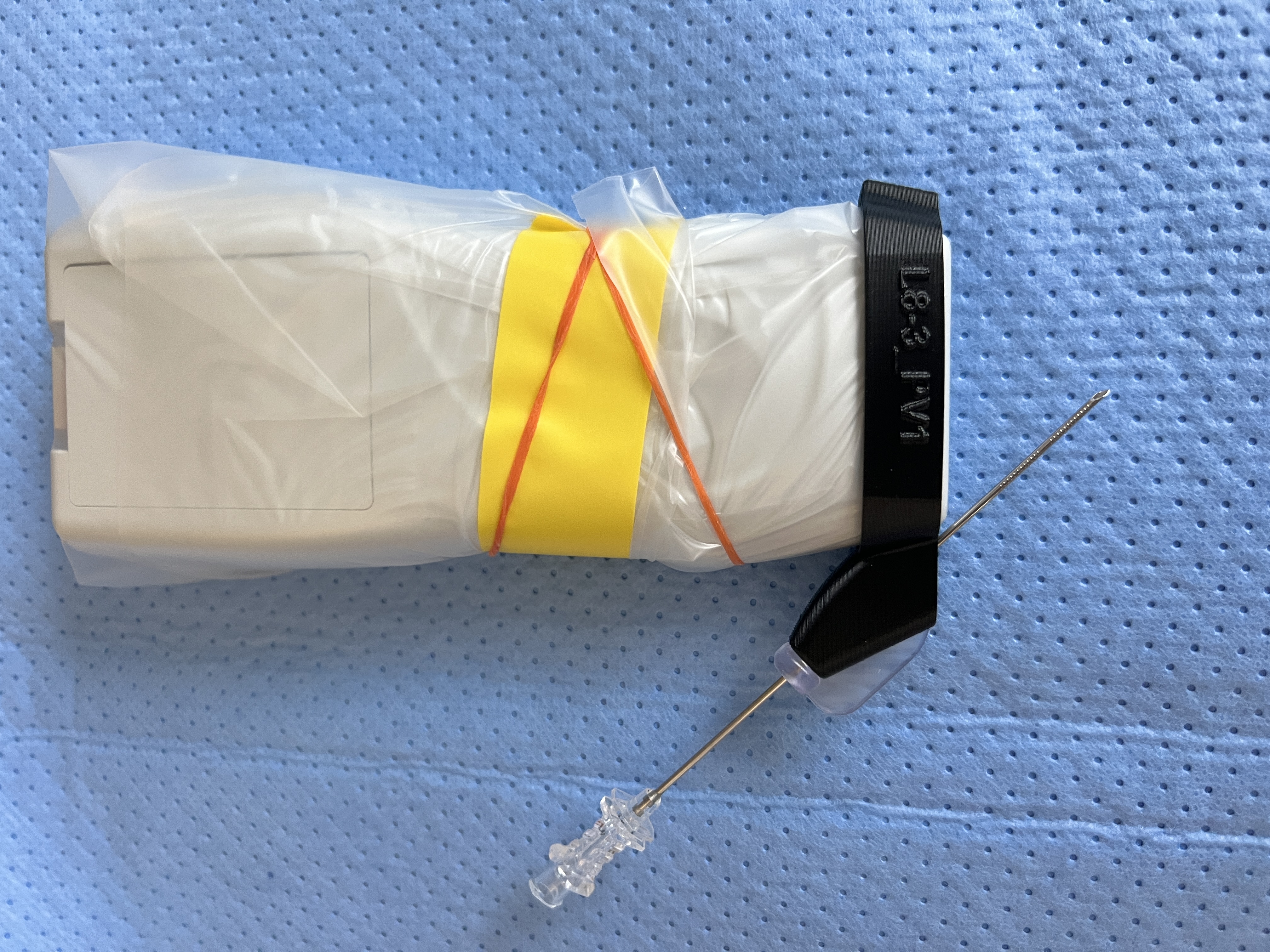

Supplement: Supplementary file 2 — Supporting Information Figure 2. Image of the transducer with the mounted needle guide. [file CCD-106-2252-s007.JPG]

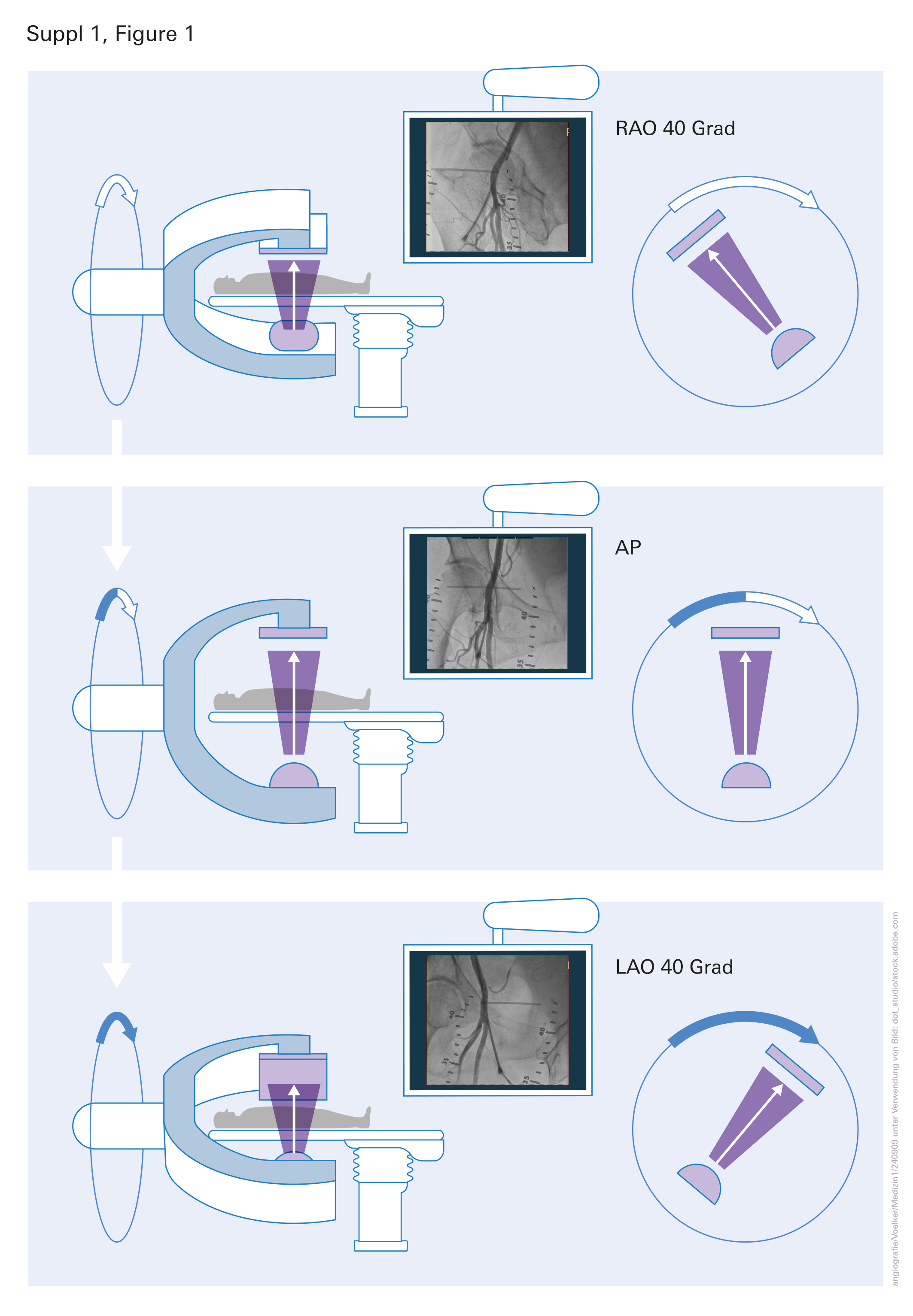

Supplement: Supplementary file 3 — Supporting Information Figure 3. Schematic illustration of rotational angiography. [file CCD-106-2252-s002.jpg]

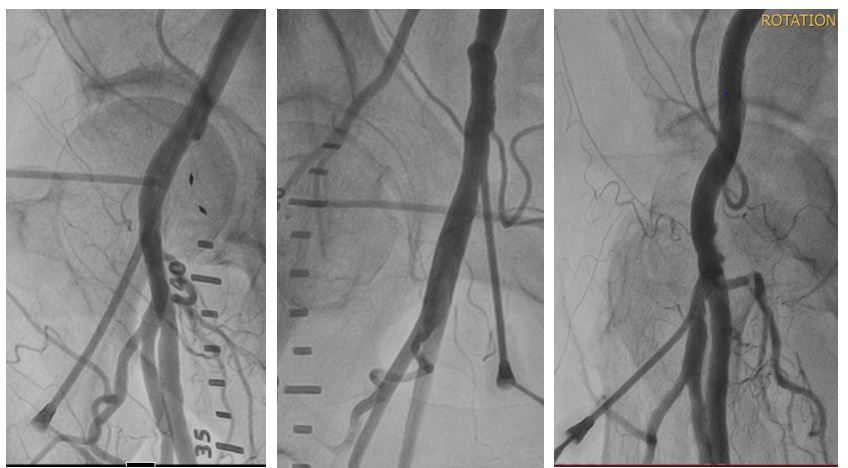

Supplement: Supplementary file 4 — Supporting Information Figure 4. Examples of proper (left) and inadequate cannulation heights. [file CCD-106-2252-s005.JPG]

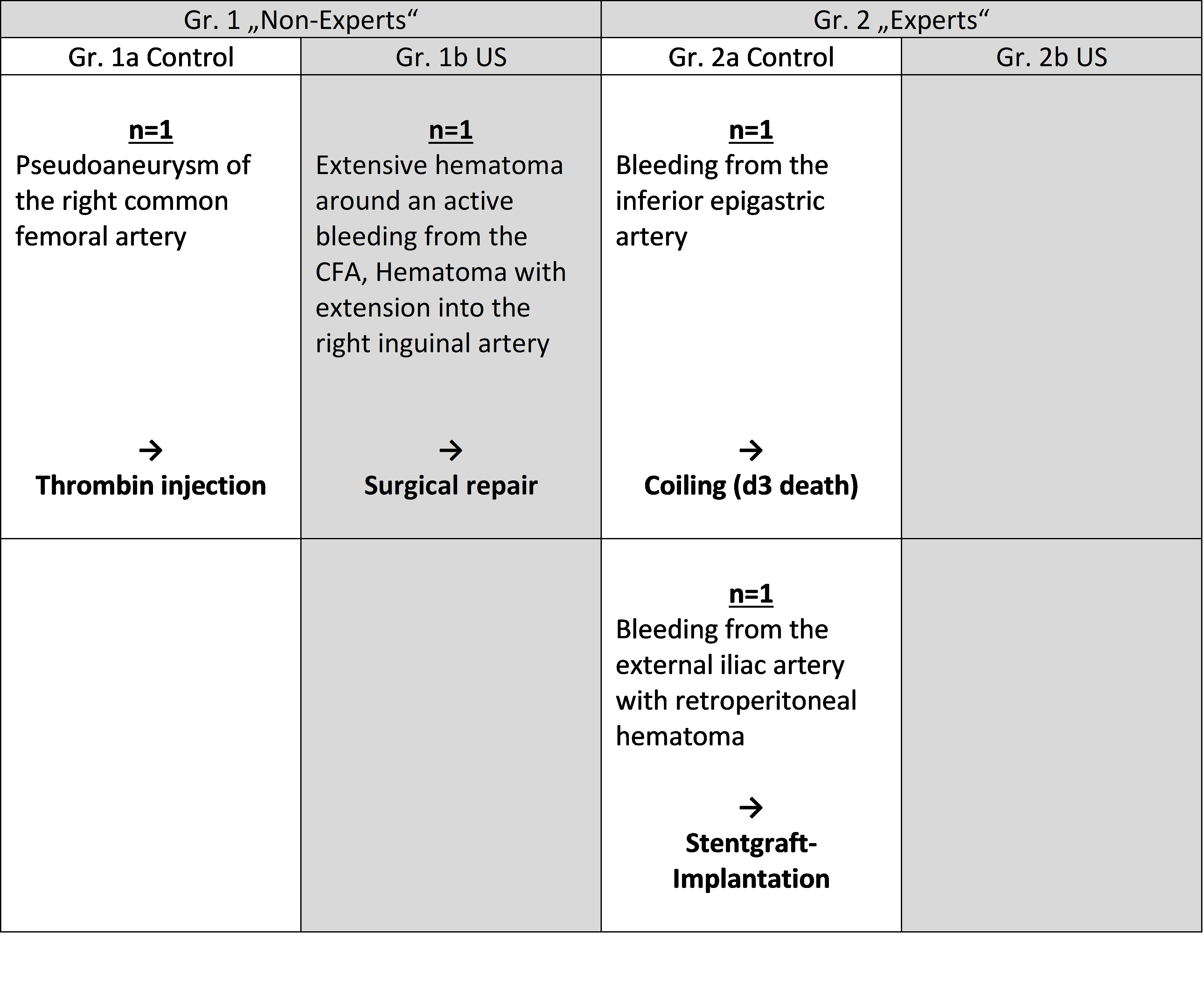

Supplement: Supplementary file 5 — Supporting Information Table 5. Clinical course of patients with bleeding complications. [file CCD-106-2252-s001.png]
