# Supplementary material for: Can Ultrasound‐Guided in‐Plane Puncture Technique Enhance the Precision of Femoral Artery Access? The Randomized PARFEM Trial
Source: Catheter Cardiovasc Interv. 2025 Jul 30;106(4):2252–62. doi: 10.1002/ccd.31733 (PMC12509261; doi:10.1002/ccd.31733)
Supplement: Supplementary file 7 — Supporting Information Appendix 7. Questionnaire on prior ultrasound experience. [file CCD-106-2252-s008.docx]

**Supporting Information Appendix 1**:

Questionnaire for study physicians on their prior experience in vascular imaging with ultrasound

***How much experience did you have at the beginning of the PARFEM study with the following ultrasound techniques (number of procedures)?***

1. Ultrasound-guided vascular imaging (e.g., carotid arteries, femoral artery, other vessels)?
   - ☐ 0
   - ☐ <20
   - ☐ 20-50
   - ☐ 50-100
   - ☐ >100
2. Ultrasound-guided vascular puncture in short axis ("out-of-plane," e.g., for central venous catheter placement)?
   - ☐ 0
   - ☐ <20
   - ☐ 20-50
   - ☐ 50-100
   - ☐ >100
3. Ultrasound-guided vascular puncture with needle guidance ("in-plane," e.g., for TAVI)?
   - ☐ 0
   - ☐ <20
   - ☐ 20-50
   - ☐ 50-100
   - ☐ >100

**Considering the responses to the questionnaire, the study physicians were categorized into two:** **ultrasound competency levels**

Level 1 (n=15): Skills in the use of ultrasound for puncture guidance (> 20 procedures, mostly ultrasound-guided central venous catheterization using the short-axis technique).

Level 2 (n=8): Experience in ultrasound-guided in-plane puncture of the femoral artery (> 20 procedures).
