# Supplementary material for: Can Ultrasound‐Guided in‐Plane Puncture Technique Enhance the Precision of Femoral Artery Access? The Randomized PARFEM Trial
Source: Catheter Cardiovasc Interv. 2025 Jul 30;106(4):2252–62. doi: 10.1002/ccd.31733 (PMC12509261; doi:10.1002/ccd.31733)
Supplement: Supplementary file 8 — Supporting Information Appendix 8. Influence of ultrasound experience on primary endpoint. [file CCD-106-2252-s006.docx]

**Supporting information Appendix 2:**

Influence of prior ultrasound experience on the primary endpoint

Subgroup analysis examined whether the level of ultrasound competence of the study physicians (regardless of their catheter experience) had an impact on the study results. A total of 122 procedures were performed by “Level 2”-examiners, while the remaining 164 procedures were performed by “Level 1”-examiners. The impact of ultrasound skills on the primary endpoint (primary successful CFA cannulation) was not significantly different for the two ultrasound skill levels (level 1: 60.9→79.2%, level 2: 47.4→81.5%) (see **Figure 4**).

Thus, this subgroup analysis revealed that examiners with no prior experience with the in-plane puncture technique benefited from ultrasound guidance to the same extent as examiners with prior experience with this puncture technique. This suggests that novices do not face a relevant learning curve, as they do with out-of-plane puncture techniques,^29^ to achieve reliable results.
